# Computational analyses of curcuminoid analogs against kinase domain of HER2

**DOI:** 10.1186/1471-2105-15-261

**Published:** 2014-08-03

**Authors:** Wannarat Yim-im, Orathai Sawatdichaikul, Suwanna Semsri, Natharinee Horata, Wanwimon Mokmak, Sissades Tongsima, Apichart Suksamrarn, Kiattawee Choowongkomon

**Affiliations:** Genetic Engineering Interdisciplinary Program, Graduate School, Kasetsart University, 50 Ngam Wong Wan Rd, Chatuchak, Bangkok, 10900 Thailand; Institute of Food Research and Product Development, Kasetsart University, 50 Ngam Wong Wan Rd, Chatuchak, Bangkok, 10900 Thailand; Faculty of medical technology, Huachiew Chalermprakiet University, Samut, Prakarn, 10540 Thailand; National Center for Genetic Engineering and Biotechnology, 113 Thailand Science Park, Phahonyothin Road, Khlong Nueng, Khlong Luang, PathumThani, 12120 Thailand; Department of Chemistry and Center of Excellence for Innovation in Chemistry, Faculty of Science, Ramkhamhaeng University, Bangkok, 10240 Thailand; Department of Biochemistry, Faculty of Science, Kasetsart University, 50 Ngam Wong Wan Rd, Chatuchak, Bangkok, 10900 Thailand; Center for Advanced Studies in Tropical Natural Resources, National Research University-Kasetsart University, Kasetsart University, Chatuchak, Bangkok, 10900 Thailand

**Keywords:** HER2, Tyrosine kinase, Curcuminoid analogs, Docking, Molecular dynamics simulation

## Abstract

**Background:**

Human epidermal growth factor receptor 2 (HER2) has an important role in cancer aggressiveness and poor prognosis. HER2 has been used as a drug target for cancers. In particular, to effectively treat HER2-positive cancer, small molecule inhibitors were developed to target HER2 kinase. Knowing that curcumin has been used as food to inhibit cancer activity, this study evaluated the efficacy of natural curcumins and curcumin analogs as HER2 inhibitors using *in vitro* and *in silico* studies. The curcumin analogs considered in this study composed of 4 groups classified by their core structure, β-diketone, monoketone, pyrazole, and isoxazole.

**Results:**

In the present study, both computational and experimental studies were performed. The specificity of curcumin analogs selected from the docked results was examined against human breast cancer cell lines. The screened curcumin compounds were then subjected to molecular dynamics simulation study. By modifying curcumin analogs, we found that protein-ligand affinity increases. The benzene ring with a hydroxyl group could enhance affinity by forming hydrophobic interactions and the hydrogen bond with the hydrophobic pocket. Hydroxyl, carbonyl or methoxy group also formed hydrogen bonds with residues in the adenine pocket and sugar pocket of HER2-TK. These modifications could suggest the new drug design for potentially effective HER2-TK inhibitors. Two outstanding compounds, bisdemethylcurcumin (AS-KTC006) and 3,5-bis((E)-3,4-dimethoxystyryl)isoxazole (AS-KTC021 ),were well oriented in the binding pocket almost in the simulation time, 30 ns. This evidence confirmed the results of cell-based assays and the docking studies. They possessed more distinguished interactions than known HER2-TK inhibitors, considering them as a promising drug in the near future.

**Conclusions:**

The series of curcumin compounds were screened using a computational molecular docking and followed by human breast cancer cell lines assay. Both AS-KTC006 and AS-KTC021 could inhibit breast cancer cell lines though inhibiting of HER2-TK. The intermolecular interactions were confirmed by molecular dynamics simulation studies. This information would explore more understanding of curcuminoid structures and HER2-TK.

**Electronic supplementary material:**

The online version of this article (doi:10.1186/1471-2105-15-261) contains supplementary material, which is available to authorized users.

## Background

Human Epidermal Growth Factor Receptor 2 (HER2) is one of the tyrosine kinase receptors in EGFR family, which includes EGFR/ErbB1, HER2/ErbB2, HER3/ErbB3 and HER4/ErbB4 [1]. Since there is no natural ligand specific to HER2, HER2 tends to form heterodimer with other ligand-induced members [2]. After dimerization, the complex can trigger downstream pathways such as Ras/Raf/MAPK and PI3K/AKT pathways to increase cell growth, cell survival and cell differentiation [3, 4]. Considerable evidencesshowed that HER2 over expression was involved in many types of cancer such as breast, ovarian, gastric and prostate cancers [5]. Therefore, HER2 is considered as a drug target for cancer therapy focusing on inhibiting HER2 to reduce tumor growth.

At present, there are two main approaches used to inhibit HER2, namely; monoclonal antibodies such as Trastuzumab, and small molecule inhibitors such as Lapatinib [6] and SYR127063 (called SYR for short) [7] targetingon tyrosine kinase domain (HER2-TK). Although Trastuzumab can downregulate HER expression, cardiotoxicity and drug resistance can be found in Trastuzumab-treated patients [8, 9]. Moreover, side effects such as diarrhea, rash or nausea can be observed in Lapatinib treatment [10]. Hence, new inhibitors are urgently required for HER2-overexpressed cancer treatment.

Recently, in 2011, the first HER2-TK structure complex with pyrimidine compound SYR was released (PDB access 3PP0), providing the new understanding of the kinase structure [7]. Unlike the active- or inactive-conformations of EGFR-TK, HER2-TK configuration was somewhat in the middle of these typical conformations. It was named “*the active-like conformation*”, due to, the orientation of the helix-αC-out, the DFG-in and unformed secondary structure of the activation loop. The second crystal of HER2-TK complex with TAK-285 (PDB access 3RCD) adopted the similar conformation as mentioned above [11].

Curcumin (also known as diferuloylmethane) is generally found as the major compound in rhizomes of turmeric plants; *Curcuma longa* Linaeusas yellow residue. It has been used as spice and ingredients in folk medicinal remedies in many Asian countries. The curcumin and its three natural analogs, curcumin II (demethoxycurcumin), curcumin III (bisdemethoxycurcumin) and cyclocurcuminpossess the remarkable pharmacological effects for centuries, such as anti-inflammatory [12, 13], antioxidant [14], anti-carcinogenesis [15–18]. Moreover, curcumins is safe to use in high dose with non-toxic report [19, 20]. Despite many advantages of curcumins, the poor stability and bioavailability profiles of curcumins are questionable when it comes to directly using crude curcumin as the potent and selective cancer drug. Many researchers have been focusing on the developing the curcumin analogs to enhance their stability and bioavailability. In particular, the novel series of curcumin-analog compounds have been synthesized and studied their effect in various cell targets [21–26]. They possess several properties, potent activity against parasite in *Trypanosoma* and *Leishmania* species [21], antimycobacterial activity [22], inhibiting nitric oxide production from Lps-activated microglial cells [25] and estrogenic properties [23, 24, 26]. Thus, in this paper, we aimed to investigate the effect of this set of curcumin analogs on the HER2-TK activity using both experimental and computational methods.

Curcumin has been shown to inhibit cancer growth by means of inhibiting several tyrosine kinases including EGFR, HER2, MAPK, phosphorylase kinase, pp60c-src tyrosine kinase, protein kinase C, and protein kinase A [18, 27–34]. Furthermore, the curcumins can inhibit various types of cancer including breast cancer cells [15, 28] and also induce the internalization of HER2 from cell surface [35]. Recently, curcumin analog cyclohexanone has shown to selectively inhibit tyrosine kinase domain of EGFR, *in vitro*, *in vivo* and *in silico* studies [36] which reveals an opportunity for direct binding between curcumins and tyrosine kinase domains of other EGFR family members. Furthermore, the *in silico* screening of the natural database against HER2 kinase showed that such curcumins could interact with kinase through benzene rings for hydrophobic interactions and carboxyl groups for hydrogen bond formation [37].

In this study, we investigated interactions between curcumin analogs and HER2-TK by using virtual screening based on molecular docking in order to find potential compounds against HER2-TK. The hit compounds have been validated by different inhibitions between two types of breast cancer cell-lines with both HER2-overexpression and HER2-non-overexpression. Such findings might be useful for further development of curcumins as a new HER2 inhibitor in the future.

## Methods

### Computational procedures

#### The preparation of ligand

The two dimensional (2D) structure of 143 curcuminoid analogs were collected from the previous studies [21–26] (Additional file 1: Table S1). The ionization states, tautomers, stereochemistries, and ring conformations of all curcuminoid structures were calculated and OPLS-2005 force field was applied using LigPrep module in Schrödinger package. These structures were used as an initial material during computational docking procedure to study interactions with the binding site of the HER2 tyrosine kinase domain.

### The preparation of protein

The atomic coordinate of HER2 tyrosine kinase domain (HER2-TK) was obtained from the crystallographic structure, accession no. 3PP0 in Protein Data Bank (PDB) [7]. This structure contains asymmetric dimer of HER2-TK complex with selective inhibitor HER2-TK, pyrrolo[3,2-d]pyrimidine-based potent, SYR. In order to perform the docking calculations, only chain A was selected as the target template. Another chain of HER2-TK as well as the co-crystalized ligand(s) and crystal water molecules were removed. Hydrogen atoms were assigned and parameterized with Optimized Potential for Liquid Simulation version 2005 (OPLS-2005 force field) using the protein preparation wizard, which continuously minimized the whole structure by the Impref module in the Schrödinger package.

### Docking procedure using Glide standard precision mode (SP mode)

The structures of protein and ligands were prepared as previously described. The OPLS-2005 force field was applied to both protein and ligands. The complexes of HER2-TK and each curcuminoid, including the co-crystal ligand were generated with molecular docking approach using Grid-based Ligand Docking with Energetics (Glide)with standard precision mode (SP mode) [38, 39]. The grid map was generated in Receptor Grid Generation by setting the center of the grid map around the catalytic site. Self-docking between HER2-TK and SYR was performed to validate all parameters before being applied to the study of interactions between HER2 and curcumins.

### Post-docking analysis

In order to handle considerable number of docking results, the sub-groups of modified core structure of curcuminoids were classified. Top ranks docking score of each sub-group were selected to further test in cell-based assay. In addition, the poor scores of each curcumin sub-groups were also chosen to be the control set in breast cancer cell-line assay.

### Molecular interaction and stability in binding pocket

All simulation steps were performed using the SANDER module of the AMBER 12 package and AMBER FF03 force-field parameters [40]. The partial atomic charges of ligand were computed by using AM1-BCC method as implemented in the Antechamber module of the AMBER package. Their atom types and missing force field parameters were assigned based on the general AMBER force field (GAFF). Each complex was immersed in an isomeric truncated-octahedron box of TIP3P water molecules (10 Å from the solute surface) and neutralized by additional Cl^-^ anions. The system was then minimized with the five-step procedure (Additional file 1: Table S2). All steps included 5,000 steepest-descent minimization cycles and 5,000 conjugate-gradient minimization cycles with different restraints on the protein structure. For the first step, harmonic restraints with a force constant of 5 kcal/(mol · Å^2^) were used to immobilize the heavy atoms of protein coordinates, excluding hydrogen atoms, at the starting positions, while solvent molecules were allowed to relax the unfavorable contacts with other solvent and solute molecules. For the second, third and fourth steps, harmonic restraints with force constants of 5, 1 and 0.5 kcal/(mol · Å^2^), respectively, were used to restrain the backbone of the protein. In the last step, the entire system was minimized with no positional restraints.

With weak positional restraints on the protein (force constant of 5 kcal/(mol · Å^2^)), all systems were heated from 0 to 300 K during a 200 ps MD simulations. After removing the restraints from the protein, we equilibrated the system with constant volume and set the constant temperature at 300 K for 500 ps. Note that we observed the equilibrium of energy (potential, kinetic and total energy), temperature, pressure, volume, density and RMSD before moving on to the production runs. The production MD simulations were performed from 30 ns while maintaining constant pressure and temperature. With a collision frequency of 1 ps^-1^, the temperature in all simulations was controlled by Langevin dynamics. Using an isotropic position scaling algorithm with a relaxation time of 2 ps, the pressure in NPT simulations was maintained at an average pressure of 1 atm. The random number generator was reseeded [41] for every simulation. A cut-off of 10 Å and the particle mesh Ewald method were employed with the default parameters to calculate long-range non bonded interactions. With the tolerance parameter of 10^-5^ Å, SHAKE constraints [42] were used to eliminate bond-stretching freedom for all bonds involving hydrogen, thereby allowing the use of a 2 fs time step. To monitor the stabilities of all systems, the Cα root-mean-square deviations (RMSD) were calculated. The RMSD of binding residues within 5 Å of the inhibitor were examined. The ptraj modules in the AMBER software were used to calculate the hydrogen bond occupancy and hydrogen bond distance between inhibitors and proteins [43, 44]. All MD simulations were calculated on 22-node Linux High Performance Computer Cluster with 32 cores of AMD 2.2 GHz.

The energy calculations were done as implemented in the MMPBSA.py script in AmberTools. The MM-PBSA approach is an acceptable method to compute the free energies of binding of ligands to proteins or to estimate the absolute free energies of molecules [43, 44]. One hundred frames from the last 5 ns of each 30 ns MD studies were selected for the analysis of ligand binding energies, sampled at 50 ps intervals. Binding free energy was estimated from each energy terms as following equations (equation 1–4),
1234

‹∆G_MMPBSA_› is referred to final calculated MM-PBSA binding energy. It is described by the difference of ΔG_complex_ by the summation of ΔG_protein_ and ΔG_ligand_ (1). The free energy of each molecular system is given by the expression in the terms of equation (2). ‹∆E_MM_› is the total molecular mechanics energy in the gas phase, ‹∆G_solv_› is a correction term (solvation free energy) of each system surrounded by solvent, and ‹T∆S› is the entropy. ‹∆E_MM_› includes electrostatic ‹∆_Eele_›, and van der Waals ‹∆E_vdw_› energies, while ‹∆G_solv_› is the sum of electrostatic solvation energy ‹∆G_pb_›, and the non-electrostatic solvation component ‹∆G_np_› (3–4). The polar contribution is calculated using PB model, while the non-polar energy is estimated by solvent accessible surface area (SASA). In this study, ‹T∆S› term was excluded.

### Experimental procedures

#### Proliferation and viability assay

Cell proliferation and viability were measured by tetrazolium 3-(4, 5-dimethylthiazol-2-yl)-2, 5-diphenyltetrazolium bromide (MTT) assay. The reaction was catalyzed by mitochondrial succinate dehydrogenease and requires NADH, which must be supplied by living cells. SKBR3 and MCF7 cell line were seeded in flat-bottomed 96-well tissue culture plates as 1 × 104 cells/well/100 μL and cultured overnight. Pure curcumin extracts and its analogs with different concentrations were dissolved in 100 μL 10% FBS-RPMI 1640 medium and added into the cells and incubated for 48 h. Then 100 μL of medium was removed and 10 μL of MTT dye (Sigma-Aldrich; USA) was added, followed by 4hourof incubation. Subsequently, the supernatant from each well was aspirated off, leaving the purple form azan crystals. Optical density was measured by an ELISA micro plate reader at 540 nm with a reference wavelength of 630 nm. Percentage of cell survival was calculated by the formula below. Each assay was done in triplicate and the standard deviations were calculated.


Absorbance of vehicle control well.

All curcumin analog compounds were synthesized and published [21–26] by the laboratory of Prof. Dr. Apichart Suksamran, Department of Chemistry and Center of Excellence for Innovation in Chemistry, Faculty of Science, Ramkhamhaeng University.

## Results and discussion

### Selection of the curcumin analogs and structure analyses

**Figure 1:**
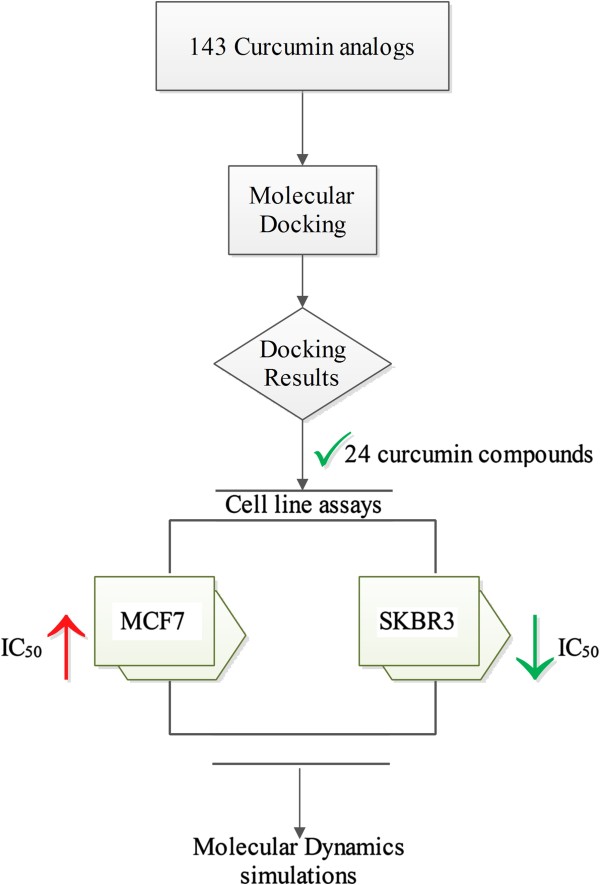
**The rational workflow of this study, starting from** ***in silico*** **screening based on molecular docking and validated with cell line assays (SKBR3:a breast cancer cell line which over-expresses the HER2 gene product, MCF-7: a breast cancer cell line that absence of HER2 protein overexpression) and deep interaction study by molecular dynamic simulation.**

**Figure 2:**
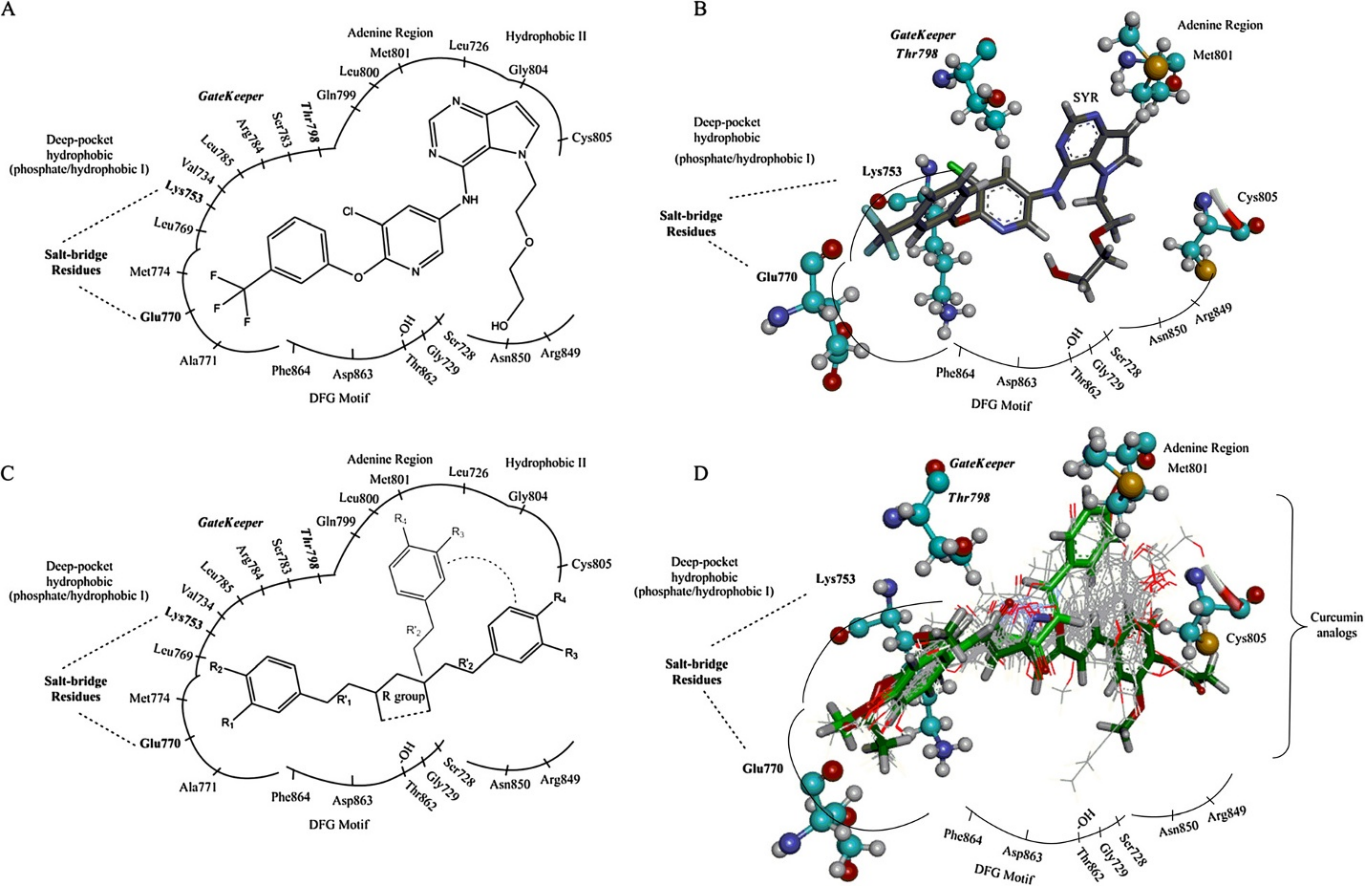
**The illustration of A) two dimensional (2D) structure and B) three dimensional (3D) structure of SYR from the x-ray structure 3PP0, while panel C) and D) present 2D and 3D structures of curcumin analogs in the binding pocket of HER2-TK from the docking results.**

Considering to the core structures of all 143 curcuminoid compounds, the middle-linear seven carbon linkage between two phenyl rings can be classified into four sub-groups,β-diketone, monoketone, pyrazole (N-N heterocyclic), and isoxazole (N-O hetorocyclic). Schematic diagram of the workflow is shown in Figure 1. All 143 curcumin analogs were docked against tyrosine kinase of HER2 by Glide SP docking. A few compounds of each sub-group in the docking top ranks were selected. All 24 chosen compounds were classified into four groups based on their core structures. From Table 1, compounds AS-KTC001 to AS-KTC011 were classified into β-diketone group. Monoketone composed of two compounds, AS-KTC012 and AS-KTC013. For AS-KTC014 to AS-KTC017 were categorized as pyrazole curcumin analogs. The last group, isoxazole curcumin analogs consisted of seven molecules, AS-KTC018 to AS-KTC024. The docked conformations of all curcumin compounds were well oriented in the ATP-binding pocket of HER2-TK (Figure 2C and 2D). One phenyl-end of curcuminoids compounds oriented well in deep hydrophobic pocket, while another phenyl-end exposed to the open gate. They could form interactions either with Met801 (adenine region) or with Cys805 (hydrophobic pocket II), depending on each configuration of analog (Figure 2D). According to the revealed three dimensional structure of HER2-TK adopted the active-like conformation, the binding cave stayed in the tunnel shape rather than the opened cave as presented in EGFR-TK. This pocket possesses approximately the volume of 475 Å^3^. As the binding pocket adopted the tunnel-like shape, the curcuminoid structures are also in the linear cylinder shape that can fit into this pocket quite well. The hydrophobic-I site merged with the phosphate binding region (Ser783, Arg784, Leu785, Leu769, Gly770, Ala771, Met774 and Phe864) as the deep semi-closed site of ATP-binding pocket. This pocket is occupied by the trifluoromethyl-phenoxy fragment of SYR, the co-crystal ligand (Figure 2A and 2B). Furthermore, one of the phenyl rings and its hydrophobic substituent groups of curcuminoids compounds were perfectly fit to this deep hydrophobic pocket. The functional groups of modified middle-linear seven carbon linkage of curcurmin analogs pointed to the DFG motif, especially the adjacent residue Thr862. In addition, another phenyl-end and its hydrophobic substituent groups of curcumin analogs could span around adenine region to hydrophobic-II site (Figure 2C and 2D). This observation was found as the common interaction among curcuminoid compounds, conformed to the pyridine and amine fragments of SYR pointed to the same residues (Figure 2A and 2B). These occupancy and interactions were considered important to increase selectivity and affinity for HER2 inhibitors [45–47]. However, the curcumin analogs could not interact with Met801 as any other EGFR/HER2-TK inhibitors, which contain 4-anilinoquinazoline, pyrrolopyrimidine, pyrrolotriazine and cyanoquinoline cores. The carbonyl group of the linkage chain, from β-diketone or monoketoncurcumins, could form a hydrogen bond directly with one of the key residues Thr862 (Thr862 OH---OC curcumins). This interaction differed from that of SYR127063 and other HER2 inhibitors that was a water-mediated hydrogen bond between N_3_ of quinazoline and Thr862 [48]. As the direct hydrogen bond formation at this position was crucial to enhance binding affinity, N_3_ of quinazoline was modified to nitrile group to form the direct hydrogen bond [48]. These occupancy and interactions mimicked those of a heterocyclic core of HER2 inhibitor in an adenine ring of ATP [49]. All 24 selected compounds have been processed in further experiments, to investigate the bioavailability profiles of curcumin analogs on two types of human breast cancer cell lines.

**Table 1:** **Two dimensional structures of curcumin analogs and its Gscore**

Code	Structure	Dock score	Ref
AS-KTC001		-8.35	[21, 22, 25]
AS-KTC002		-8.17	[21, 22, 25]
AS-KTC003		-8.79	[21, 22, 25]
AS-KTC004		-8.24	[21, 22, 25]
AS-KTC005		-8.65	[21, 22, 25]
AS-KTC006		-8.18	[21, 22, 25]
AS-KTC007		-8.20	[21, 22]
AS-KTC008		-6.40	[21, 22]
AS-KTC009		-8.66	[21, 22]
AS-KTC010		-9.03	[21, 22]
AS-KTC011		-9.26	[21, 22]
AS-KTC012		-8.56	[21]
AS-KTC013		-8.24	[22]
AS-KTC014		-8.10	[22]
AS-KTC015		-7.71	[22]
AS-KTC016		-7.82	[22]
AS-KTC017		-8.61	[22]
AS-KTC018		-8.04	[22]
AS-KTC019		-8.67	[22]
AS-KTC020		-7.76	[22]
AS-KTC021		-7.12	[22]
AS-KTC022		-7.14	[22]
AS-KTC023		-7.35	[22]
AS-KTC024		-7.33	[22]

### The bioavailability profiles of curcumin analogs on two types of human breast cancer cell lines

The HER2-TK inhibitory assay was performed using a commercialized HER2-TK, but unfortunately, activity of the HER2-TK could not be detected (data not shown). Moreover curcumins were stable and soluble in very low pH which is not suitable for HER2-TK to stay active. Therefore, the cell-based assay was chosen instead of the purified protein based assay to investigate the inhibitory effect of curcumin analogs to HER2-TK. In order to identify the curcumin analogs against HER2-TK, two different types of human breast cancer cell lines, MCF-7 and SKBR3 were performed. The SKBR3 is a breast cancer cell line which over-expresses the HER2 gene product while the MCF-7 is in absence of HER2 protein overexpression [50–54]. Therefore, such compounds should be more effective against SKBR3 than MCF-7. The lapatinib which was tested on both breast cancer cell lines showed the IC50 on SKBR3 lower than MCF-7 [55]. Twenty-four selected compounds were determined IC_50_ on both breast cancer cell lines by MTT assay as shown in Table 2. Although most of curcumin analogs have very similar structures, each analog showed different activities on both cells. In the β-diketone group from Table 1, only AS-KTC006 showed the effective inhibiting to HER2-positive cancer cell-line (SKBR3), vice versa, lack of inhibiting to HER2-negative cancer cell-line (MCF-7). The core structure of this group composes of β-diketone which is modified substituent from natural curcumins. On the other hand none of curcumin analogs in monoketone and pyrazole groups showed selectively inhibiting activity among both cancer cell-lines. In the last group, AS-KTC021 also presented the outstanding suppress on SKBR3 but not MCF7 among isoxazole analogs. The IC_50_ for AS-KTC006 and AS-KTC021 in SKBR3 were 15.4 and 16.9 μM, respectively, and IC_50_ for both compounds in MCF7 were higher than 100 μM. Since both AS-KTC006 and AS-KTC021 were selected from docking results, they could inhibit the breast cancer cells through blocking of HER2-TK activities. Therefore, the AS-KTC006 and AS-KTC021 were chosen for further investigation the interaction mechanisms by molecular dynamics simulations.

**Table 2:** **The inhibitory activity profiles of curcumin analogs on MCF7 and SKBR3 cells**

	SKBR3	MCF7		SKBR3	MCF7
AS-KTC	IC_50_	IC_50_	AS-KTC	IC_50_	IC_50_
	(μM)	(μM)		(μM)	(μM)
001	8.3 ± 0.6	41.9 ± 12.3	013	> 100	> 100
002	13.0 ± 1.8	44.4 ± 10.8	014	10.8 ± 5.5	9.9 ± 3.5
003	24.9 ± 2.3	81.6 ± 26.0	015	30.9 ± 4.5	15.4 ± 3.8
004	> 100	79.4 ± 9.8	016	42.6 ± 5.5	33.8 ± 5.8
005	> 100	> 100	017	21.3 ± 3.8	14.3 ± 1.9
006	15.4 ± 3.9	> 100	018	33.8 ± 7.2	22.4 ± 7.7
007	7.9 ± 2.5	17.5 ± 4.5	019	> 100	38.9 ± 9.1
008	7.9 ± 2.5	36.7 ± 5.8	020	25 ± 5.5	44.1 ± 9.0
009	8.2 ± 0.4	22.1 ± 0.1	021	16.9 ± 3.4	> 100
010	> 100	> 100	022	> 100	> 100
011	> 100	> 100	023	> 100	24.3 ± 8.7
012	9.9 ± 1.0	14.3 ± 1.6	024	> 100	> 100

### Molecular interaction, stability binding free energy via MM-PBSA

**Figure 3:**
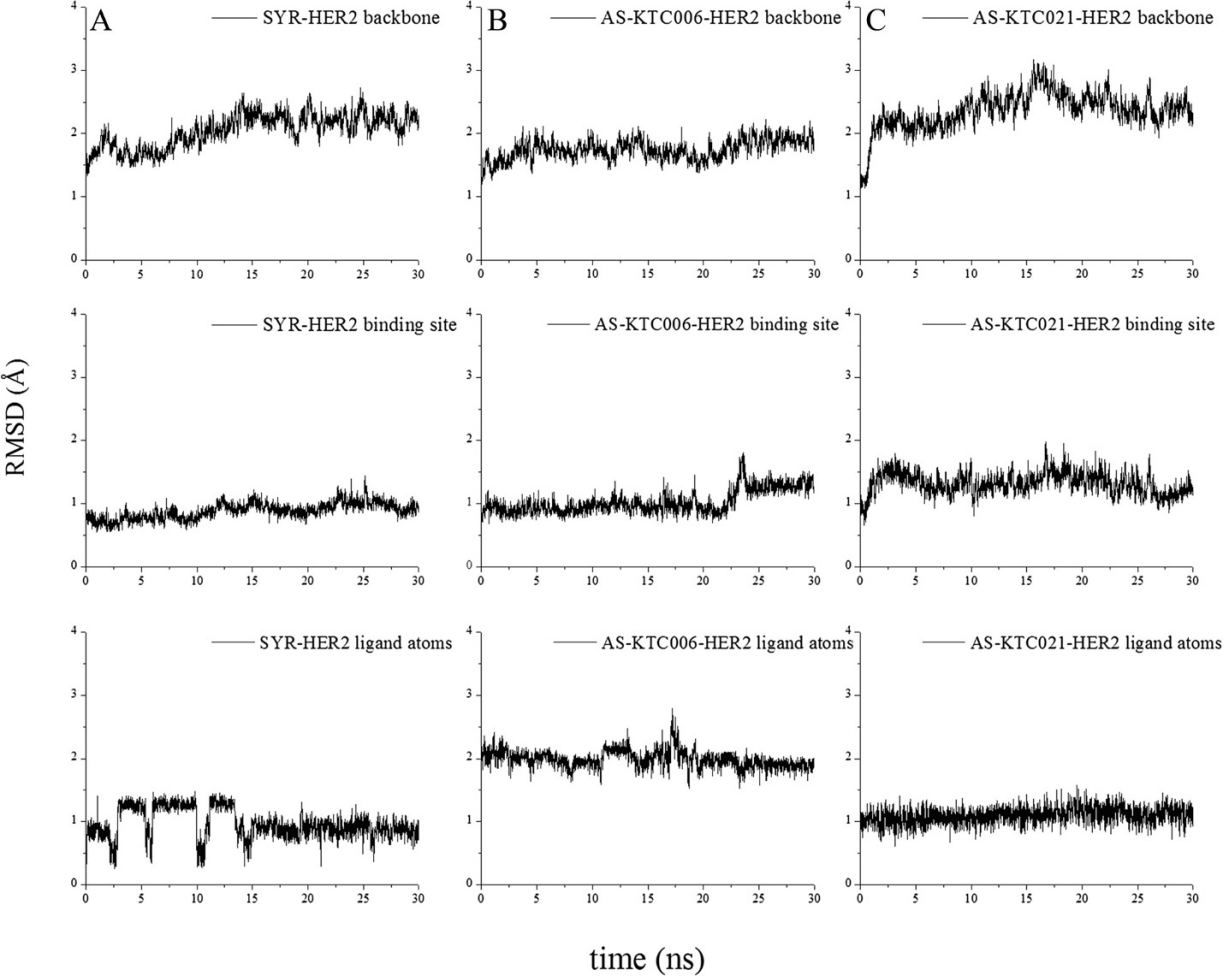
**RMSD plots of each MD simulations, presenting the backbone (N, O, C and Cα atoms of HER2-TK, the binding residues atoms of HER2-TK and ligand atoms in upper, middle and lower rows, respectively, in the panel of A) SYR B) AS-KTC006 and C) AS-KTC021.**

**Figure 4:**
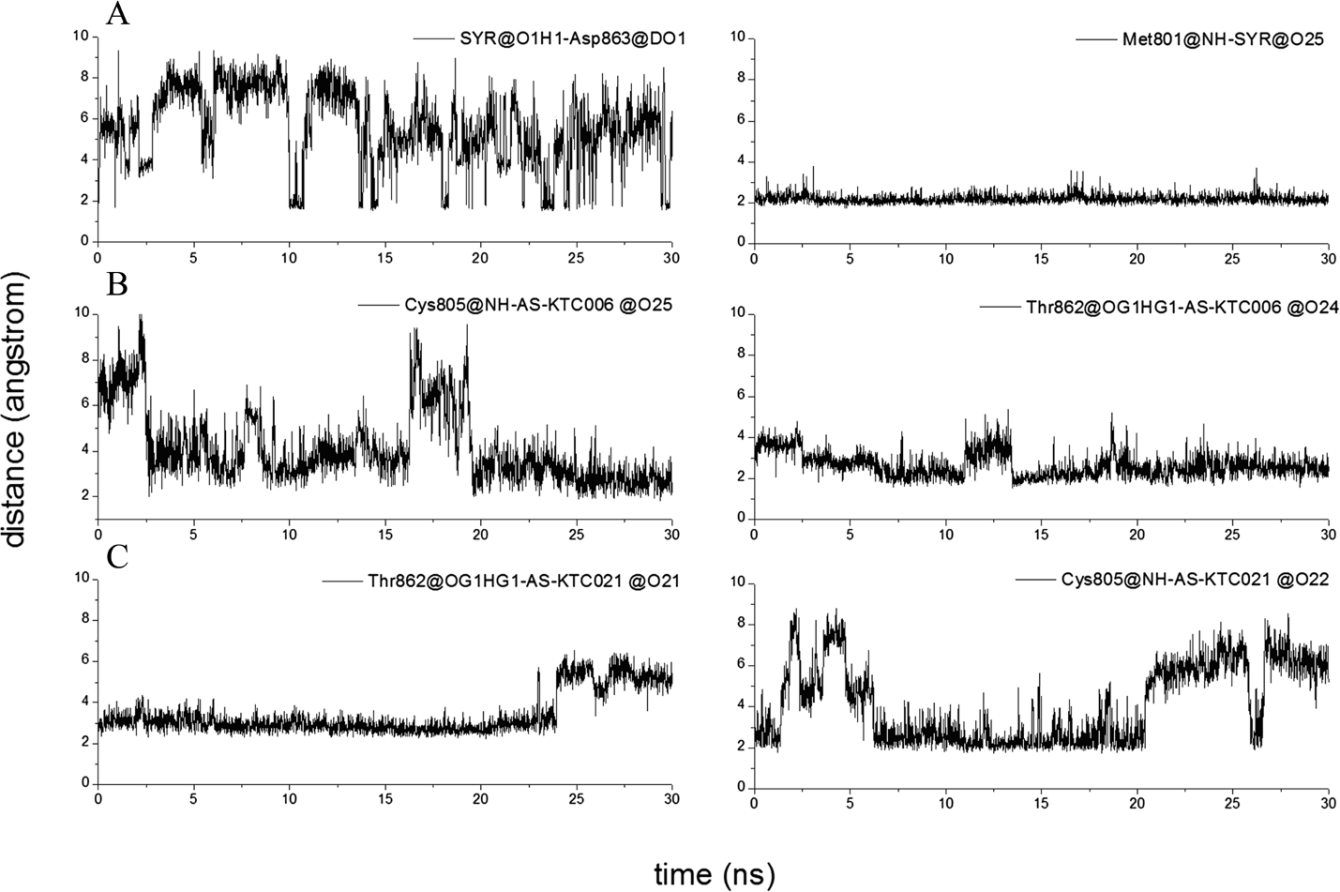
**Distance between the pairwise atoms of H-bond analyses, A) SYR-HER2TK, B) AS-KTC006-HER2TK, and C) AS-KTC02-HER2TK.**

The molecular dynamics (MD) simulations were performed to examine the molecular interaction of the bothAS-KTC006 and AS-KTC021 curcuminoids in the ATP-binding pocket of HER2-TK. As mention in the previous section, the x-ray crystal structure of HER2-TK, 3PP0 has been used as the model reference template of this study. The complexes of HER2-TK-AS-KTC006 and HER2-TK-AS-KTC021 which were constructed using molecular docking procedure have been used as starting coordinates for MD calculations. The root mean square deviations (RMSD) of all systems (backbone atoms, ligand atoms and binding site atoms) seemed to fit nicely in the binding pocket of HER2-TK (Figure 3, Additional file 1: Table S3). The structure of curcuminoid distinguishes from other known tyrosine kinase inhibitors, which generally containing either quinazoline or pyrrolopyrimidine based structures [56]. The structures of curcumin analogs adopted the long thread with two knots at each end, resulting in freely flexible structure in the tunnel-like binding pocket. Interestingly, the MD results revealed that the hydrogen bonds between H-N atom of Met801 (located on adenine region) and N-atom at position N11 of SYR existed about 94.73% along the entire 30 ns of simulation time. On the other hand, the curcumin analogs possess the interesting hydrogen bond pairs in both AS-KTC006 and AS-KTC021. Rather than forming hydrogen bond with Met801, the β-diketone curcumin analog (AS-KTC006) formed hydrogen bonds to Thr862, Cys805 and Asp863, in the binding pocket of HER2-TK. The formation with these three amino acids existed approximately 34.67%, 10.17% and 9.17%, respectively along the entire MD simulations (Table 3). Distances chromatograms of each pairwise atom from H-bond analyses were shown in Figure 4. One of these three residues is DFG-motif (Asp863), and another is adjacent residue of DFG-motif (Thr862). For AS-KTC021 (isoxazole curcumin analog), the hydrogen boding with Cys805 and Thr862 existed about 35.97% and 5.37%, respectively, along the entire MD simulations. As illustrated in Figure 2C and 2D, one of the phenyl-end of curcumin compounds were well oriented in the ATP-binding pocket of HER2-TK, while the other-end exposed to solvent giving a chance to interact with Cys805, while the linker in the middle of cucurmin structure could interact with Thr862. The binding residues of SYR-HER2-TK systems appear to be stable along the MD simulations (Figure 3A-1 and 3A-2). The system started to converge since 15 ns of simulations time (Figure 3A). In addition, the simulation systems of AS-KTC006-HER2TK and AS-KTC021-HER2TK seem to converge after 25 ns of simulations time (Figure 3). From the molecular dynamic results suggested that AS-KTC006 had better binding affinity with HER2-TK than that of AS-KTC021. Considering at the curcuminoids structures, AS-KTC006 is more flexible than AS-KTC021. In particular, AS-KTC0006 possesses β-diketonemoiety on both sides providing the flexibility of the molecule and allowing O-atom of ketone to interact with Thr862. The binding energy calculations were performed to further understand the interactions of each system. It is to be noted that protein–ligand entropy contributions were not included in these free energy values since the present MM-PBSA are typically time consuming and unreliable. Considering the intermolecular interaction of the ligands with HER2-TK in contribution terms (Table 4), non-polar contributions, the summation of ΔE_VDW_ and ΔG_np_, are significant with all ligand(s)-HER2TK systems. Interestingly, ligand AS-KTC006 shows the most favorable electrostatic interaction with the binding pocket of HER2TK. There are the agreement between MM-PBSA binding energy calculations, docking and MD simulations. The consistent observations were presented in all three different computational approaches, namely, focusing on O-atom of ketone to interact with Thr862 of the system AS-KTC006-HER2TK complex. Recently, the computational model of anti-HER2 ligands, the analogous of 4-anilinoquinazoline were reported [57, 58]. These works showed that the vdW term could be a major factor of the ligand-protein interactions; hence, the deep hydrophobic pocket would be the selectivity pocket of HER2-TK [57, 58]. Rather than focusing on the 4-anilinoquinazoline core structure (the known HER2-TK inhibitors), we focused on the curcuminoid core structure in this study. Both selected curcumin analogs form the distinguish interaction moiety from the known inhibitors of HER2-TK. Furthermore, they occupied well in the deep hydrophobic pocket of HER2-TK.

**Table 3:** **Conclusion of H-bonds between compounds and tyrosine kinase of HER2**

System	Donor			Acceptor		% occupied	distance (Å)
SYR-HER2TK	Met801	N	H	SYR	N11	94.73	3.128 ± 0.15
	SYR	O1	H1	Asp863	OD1	10.67	2.761 ± 0.17
AS-KTC006-HER2TK	Thr862	OG1	HG1	AS-KTC006	O24	34.67	2.851 ± 0.18
	Cys805	N	H	AS-KTC006	O25	10.17	3.191 ± 0.18
	AS-KTC006	O21	H39	Asp863	O	9.17	2.758 ± 0.16
AS-KTC021-HER2TK	Cys805	N	H	AS-KTC021	O22	35.97	3.142 ± 0.18
	Thr862	OG1	HG1	AS-KTC021	O21	5.37	3.100 ± 0.20

**Table 4:** **Individual terms of MM-PBSA binding energy (kcal mol** ^**-1**^ **), entropy term excluded**

System	‹∆E _vdw_›		‹∆E _ele_›		‹∆G _pb_›		‹∆G _np_›		‹∆G _solv_›		‹∆G _MMPBSA_›		Nonpolar/hydrophobic		Polar/electrostatic	
SYR-HER2TK	-67.85	(0.32)	-16.38	(0.53)	45.48	(0.44)	-43.74	(0.13)	1.73	(0.46)	-82.50	(0.45)	-111.59	(0.45)	29.10	(0.97)
AS-KTC006-HER2TK	-42.81	(0.28)	-57.70	(0.46)	76.27	(0.37)	-34.02	(0.08)	42.25	(0.37)	-58.26	(0.39)	-76.83	(0.36)	18.57	(0.83)
AS-KTC021-HER2TK	-51.24	(0.30)	-18.15	(0.63)	37.15	(0.63)	-34.75	(0.16)	2.41	( 0.60)	-66.98	(0.38)	-85.99	(0.46)	19.00	(1.26)

‹∆E_vdw_› and ‹∆E_ele_› - van der Waals and electrostatic contributions to binding energy.

‹∆G_pb_› and ‹∆G_np_› - electrostatic and nonpolar contributions to the solvation free energy.

‹∆G_MMPBSA_› - final calculated MM-PBSA binding energy.

Nonpolar contribution = ∆E_vdw_ + ∆G_np_; polar contribution = ∆E_ele_ + ∆G_pb._

## Conclusion

In the present study, we screened a series of curcumin compounds using a computational molecular docking. Then, the bioavailability assay of curcumin analogs, were conducted on two types of human breast cancer cell lines to select the specific active HER2 kinase inhibitors. The results suggested that bisdemethylcurcumin compound (AS-KTC006, CAS no. 60831-46-1) and 3,5-bis((E)-3,4-dimethoxystyryl)isoxazole (AS-KTC021, CAS no. 1118765-46-0) could inhibit breast cancer cell lines though HER2-TK. In addition, the intermolecular studies from MD simulation suggested that both selected curcumin analogs form the distinguish interaction moiety from the known inhibitors of HER2-TK. MM-PBSA binding calculation suggested that non-polar contributions are not only significant with all ligand(s)-HER2TK systems but also a major factor of the ligand-protein interactions.

## Electronic supplementary material

Additional file 1: **Table S1-S3. Table S1.** List of 143 curcuminoids compounds, which used in this present study. **Table S2.** Explanation of each simulation steps of minimization and molecular dynamics simulations. **Table S3.** List of binding residues of each system, which mentioned in Figure 3. (DOCX 493 KB)

## Abbreviations

HER2: Human Epidermal Growth Factor Receptor 2
EGFR: Epidermal Growth Factor Receptor
HER2-TK: Tyrosine kinase domain of HER2
RMSD: Root-mean-square deviations
SYR127063: 2-{2-[4-({5-chloro-6-[3-(trifluoromethyl)phenoxy]pyridin-3-yl}amino)-5H-pyrrolo[3,2-d]pyrimidin-5- yl]ethoxy}ethanol
TAK-285: N-{2-[4-({3-chloro-4-[3-(trifluoromethyl)phenoxy]phenyl}amino)-5H-pyrrolo[3,2-d]pyrimidin-5-yl]ethyl}-3- hydroxy-3-methylbutanamide
Glide: Grid-based Ligand Docking with Energetics
SP mode: Standard precision mode
AS-KTC006: Bisdemethylcurcumin, also called Di-O-demethylcurcumin, Bis(3,4-Dihydroxy-trans-cinnamoyl)methane (CAS no. 60831-46-1)
AS-KTC021: 3,5-bis((E)-3,4-dimethoxystyryl)isoxazole (CAS no.1118765-46-0)
SKBR3: A breast cancer cell line which over-expresses the HER2 gene product
MCF-7: A breast cancer cell line that absence of HER2 protein overexpression.
