# Supplementary material for: Computational analyses of curcuminoid analogs against kinase domain of HER2
Source: BMC Bioinformatics. 2014 Aug 3;15(1):261. doi: 10.1186/1471-2105-15-261 (PMC4143557; doi:10.1186/1471-2105-15-261)
Supplement: Supplementary file 1 — Additional file 1: Table S1-S3. Table S1. List of 143 curcuminoids compounds, which used in this present study. Table S2. Explanation of each simulation steps of minimization and molecular dynamics simulations. Table S3. List of binding residues of each system, which mentioned in Figure 3. (DOCX 493 KB) [file 12859_2014_6546_MOESM1_ESM.docx]

|  | **Supplementary Table 1** List of 143 curcuminoids compounds, which used in this present study. | | |  |  |  |  |
| --- | --- | --- | --- | --- | --- | --- | --- |
|  | Group / Name | IUPAC name | Structure | Glide score (kcal/mol) | Reference | Code | Note |
|  | 1 / beta-diketone | (1E,6E)-1,7-bis(4-hydroxy-3-methoxyphenyl)hepta-1,6-diene-3,5-dione |  | -8.32 | [21, 22, 25] | AS-KTC001 | diferuloymethane (curcuminI) |
|  | 1 / beta-diketone | (1E,6E)-1-(4-hydroxy-3-methoxyphenyl)-7-(4-hydroxyphenyl)hepta-1,6-diene-3,5-dione |  | -8.17 | [21, 22, 25] | AS-KTC002 | demethoxycurcumin (curcumin II) |
|  | 1 / beta-diketone | (1E,6E)-1,7-bis(4-hydroxyphenyl)hepta-1,6-diene-3,5-dione |  | -8.79 | [21, 22, 25] | AS-KTC003 | bisdemethoxycurcumin (curcumin III) |
|  | 1 / beta-diketone | (1E,6E)-1-(3,4-dihydroxyphenyl)-7-(4-hydroxy-3-methoxyphenyl)hepta-1,6-diene-3,5-dione |  | -7.77 | [22] |  |  |
|  | 1 / beta-diketone | (1E,6E)-1,7-bis(3,4-dihydroxyphenyl)hepta-1,6-diene-3,5-dione |  | -8.18 | [21, 22, 25] | AS-KTC006 |  |
|  | 1 / beta-diketone | (1E,6E)-1-(3,4-dihydroxyphenyl)-7-(4-hydroxyphenyl)hepta-1,6-diene-3,5-dione |  | -8.14 | [22] |  |  |
|  | 1 / beta-diketone | (1E,6E)-1-(3,4-dimethoxyphenyl)-7-(4-hydroxy-3-methoxyphenyl)hepta-1,6-diene-3,5-dione |  | -8.20 | [21, 22] | AS-KTC007 |  |
|  | 1 / beta-diketone | (1E,6E)-1,7-bis(3,4-dimethoxyphenyl)hepta-1,6-diene-3,5-dione | 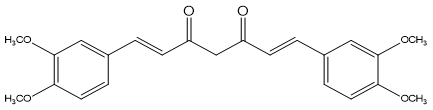 | -6.40 | [21, 22] | AS-KTC008 |  |
|  | 1 / beta-diketone | (1E,6E)-1-(4-hydroxy-3-methoxyphenyl)-7-(3-methoxy-4-propoxyphenyl)hepta-1,6-diene-3,5-dione | 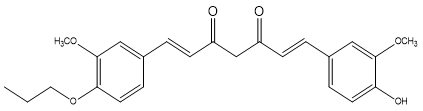 | -7.31 | [22] |  |  |
|  | 1 / beta-diketone | (1E,6E)-1-(4-hydroxy-3-methoxyphenyl)-7-(3-methoxy-4-propoxyphenyl)hepta-1,6-diene-3,5-dione |  | -8.66 | [21, 22] | AS-KTC009 |  |
|  | 1 / beta-diketone | (1E,6E)-1-(4-(allyloxy)-3-methoxyphenyl)-7-(4-hydroxy-3-methoxyphenyl)hepta-1,6-diene-3,5-dione | 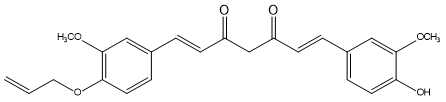 | -6.16 | [22] |  |  |
|  | 1 / beta-diketone | (1E,6E)-1-(4-hydroxy-3-methoxyphenyl)-7-(3-methoxy-4-(pentyloxy)phenyl)hepta-1,6-diene-3,5-dione | 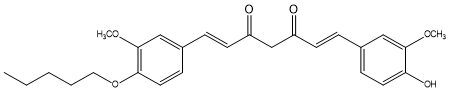 | -6.69 | [22] |  |  |
|  | 1 / beta-diketone | (E)-1,7-bis(4-hydroxy-3-methoxyphenyl)hept-1-ene-3,5-dione | 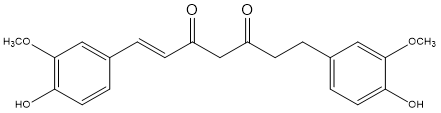 | -8.22 | [22] |  |  |
|  | 1 / beta-diketone | 1,7-bis(4-hydroxy-3-methoxyphenyl)heptane-3,5-dione | 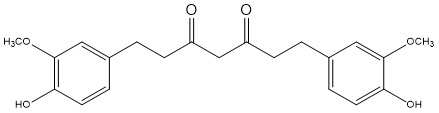 | -8.24 | [21, 22, 25] | AS-KTC004 |  |
|  | 1 / beta-diketone | 1-(4-hydroxy-3-methoxyphenyl)-7-(4-hydroxyphenyl)heptane-3,5-dione | 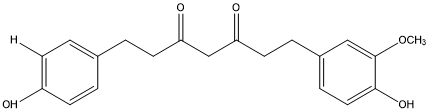 | -7.52 | [25] |  | *adopt difference configuration in 3D structure |
|  | 1 / beta-diketone | 1-(4-hydroxy-3-methoxyphenyl)-7-(4-hydroxyphenyl)heptane-3,5-dione | 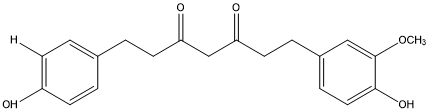 | -7.45 | [25] |  |  |
|  | 1 / beta-diketone | 1-(4-hydroxy-3-methoxyphenyl)-7-(4-hydroxyphenyl)heptane-3,5-dione | 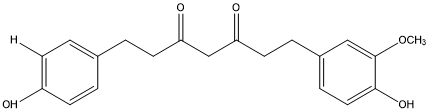 | -7.13 | [25] |  |  |
|  | 1 / beta-diketone | 1-(4-hydroxy-3-methoxyphenyl)-7-(4-hydroxyphenyl)heptane-3,5-dione | 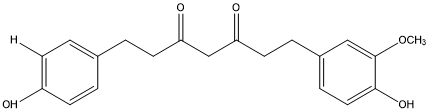 | -6.96 | [25] |  |  |
|  | 1 / beta-diketone | 1-(4-hydroxy-3-methoxyphenyl)-7-(4-hydroxyphenyl)heptane-3,5-dione | 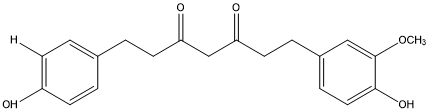 | -6.93 | [25] |  |  |
|  | 1 / beta-diketone | 1,7-bis(4-hydroxyphenyl)heptane-3,5-dione | 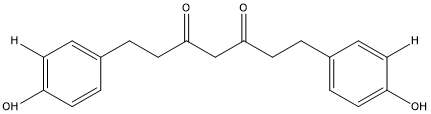 | -8.65 | [21, 22, 25] | AS-KTC005 |  |
|  | 1 / beta-diketone | (1E,6E)-1-(4-hydroxy-3-methoxyphenyl)-7-(4-methoxyphenyl)hepta-1,6-diene-3,5-dione | 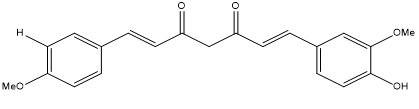 | -9.04 | [21] |  |  |
|  | 1 / beta-diketone | (1E,6E)-1-(3,4-dimethoxyphenyl)-7-(4-methoxyphenyl)hepta-1,6-diene-3,5-dione | 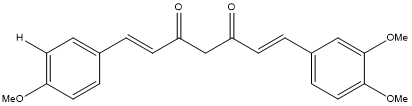 | -8.45 | [21] |  |  |
|  | 1 / beta-diketone | (1E,6E)-1-(4-hydroxyphenyl)-7-(4-methoxyphenyl)hepta-1,6-diene-3,5-dione | 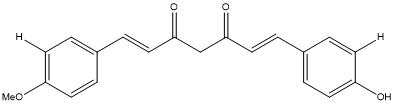 | -9.03 | [21,22] | AS-KTC010 |  |
|  | 1 / beta-diketone | (1E,6E)-1-(4-hydroxy-3-methoxyphenyl)-7-(3-methoxy-4-((3-methylbut-2-en-1-yl)oxy)phenyl)hepta-1,6-diene-3,5-dione | 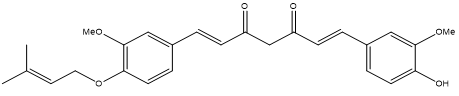 | -6.52 | [21] |  |  |
|  | 1 / beta-diketone | (1E,6E)-1,7-bis(3-methoxy-4-((3-methylbut-2-en-1-yl)oxy)phenyl)hepta-1,6-diene-3,5-dione | 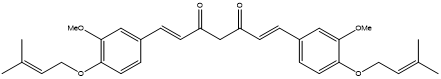 | -5.64 | [21] |  |  |
|  | 1 / beta-diketone | (1E,6E)-1-(3-methoxy-4-((3-methylbut-2-en-1-yl)oxy)phenyl)-7-(4-((3-methylbut-2-en-1-yl)oxy)phenyl)hepta-1,6-diene-3,5-dione | 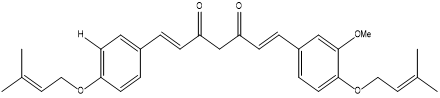 | -9.26 | [21, 22] | AS-KTC011 |  |
|  | 1 / beta-diketone | (1E,6E)-1-(4-hydroxy-3-methoxyphenyl)-7-(4-(2-hydroxyethoxy)phenyl)hepta-1,6-diene-3,5-dione | 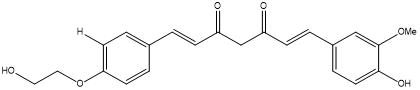 | -8.04 | [21] |  |  |
|  | 1 / beta-diketone | (1E,6E)-1-(4-(2-hydroxyethoxy)-3-methoxyphenyl)-7-(4-hydroxyphenyl)hepta-1,6-diene-3,5-dione | 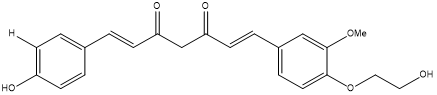 | -8.54 | [21] |  |  |
|  | 1 / beta-diketone | (1E,6E)-1-(4-hydroxy-3-methoxy-5-nitrophenyl)-7-(4-hydroxy-3-methoxyphenyl)hepta-1,6-diene-3,5-dione | 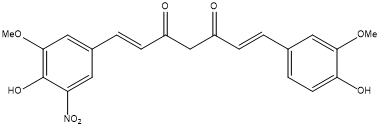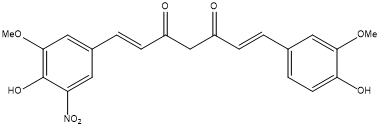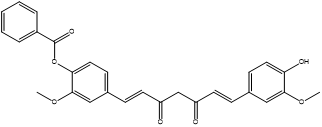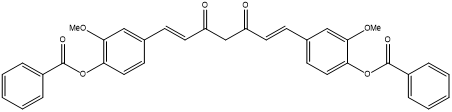   \|  \| \| --- \| | -6.62 | [21] |  |  |
|  | 1 / beta-diketone | (1E,6E)-1-(4-hydroxy-3-methoxy-5-nitrophenyl)-7-(4-hydroxy-3-methoxyphenyl)hepta-1,6-diene-3,5-dione |  | -6.49 | [21] |  |  |
|  | 1 / beta-diketone | 1,6-Heptadiene-3,5-dione, 1-[4-(benzoyloxy)-3-methoxyphenyl]-7-(4-hydroxy-3-methoxyphenyl)-, (1E,6E)- |  | -7.46 | [21] |  |  |
|  | 1 / beta-diketone | ((1E,6E)-3,5-dioxohepta-1,6-diene-1,7-diyl)bis(2-methoxy-4,1-phenylene) dibenzoate |  | -7.27 | [21] |  |  |
|  | 1 / beta-diketone | 4-((1E,6E)-7-(4-hydroxyphenyl)-3,5-dioxohepta-1,6-dien-1-yl)-2-methoxyphenyl benzoate | 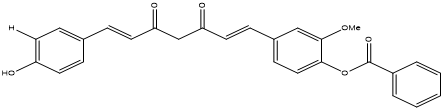 | -7.93 | [21] |  |  |
|  | 1 / beta-diketone | 4-((1E,6E)-7-(4-(benzoyloxy)-3-methoxyphenyl)-3,5-dioxohepta-1,6-dien-1-yl)phenyl benzoate | 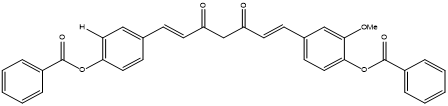 | -6.89 | [21] |  |  |
|  | 1 / beta-diketone | 4-((1E,6E)-7-(4-hydroxyphenyl)-3,5-dioxohepta-1,6-dien-1-yl)phenyl benzoate | 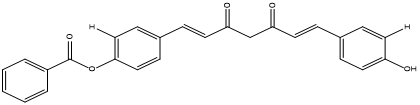 | -8.00 | [21] |  |  |
|  | 1 / beta-diketone | ((1E,6E)-3,5-dioxohepta-1,6-diene-1,7-diyl)bis(4,1-phenylene) dibenzoate | 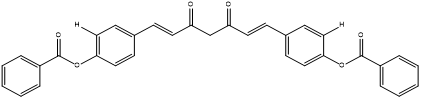 | -8.05 | [21] |  |  |
|  | 2 / mono-ketone | 5-hydroxy-1,7-bis(4-hydroxy-3-methoxyphenyl)heptan-3-one | 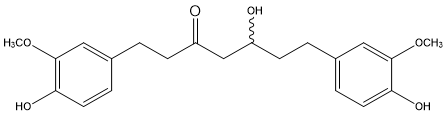 | -8.18 | [22] |  |  |
|  | 2 / mono-ketone | 5-hydroxy-1,7-bis(4-hydroxy-3-methoxyphenyl)heptan-3-one | 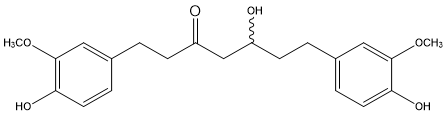 | -7.51 | [22] |  |  |
|  | 2 / mono-ketone | (E)-1,7-bis(4-hydroxy-3-methoxyphenyl)hept-4-en-3-one | 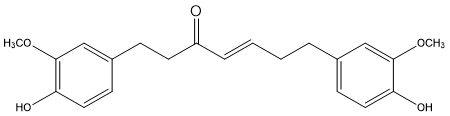 | -8.15 | [22] |  |  |
|  | 2 / mono-ketone | (1E,4E)-1,7-bis(4-hydroxy-3-methoxyphenyl)hepta-1,4-dien-3-one | 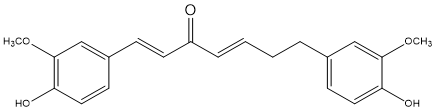 | -7.95 | [22] |  |  |
|  | 2 / mono-ketone | (4E,6E)-1,7-bis(4-hydroxy-3-methoxyphenyl)hepta-4,6-dien-3-one | 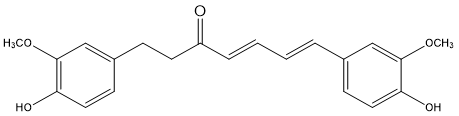 | -8.56 | [21, 22] | AS-KTC012 |  |
|  | 2 / mono-ketone | (1E,4E,6E)-1,7-bis(4-hydroxy-3-methoxyphenyl)hepta-1,4,6-trien-3-one | 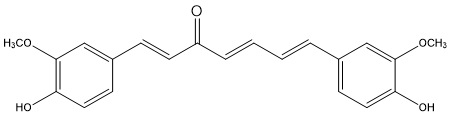 | -8.12 | [22] |  |  |
|  | 2 / mono-ketone | 1,7-bis(4-hydroxy-3-methoxyphenyl)heptan-3-one | 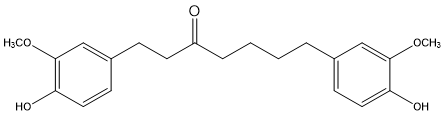 | -8.24 | [22] | AS-KTC013 |  |
|  | 2 / mono-ketone | (E)-1,7-diphenylhept-6-en-3-one | 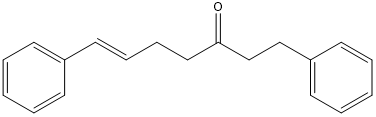 | -7.58 | [23] |  |  |
|  | 2 / mono-ketone | 5-hydroxy-7-(4-hydroxy-3-methoxyphenyl)-1-(4-hydroxyphenyl)heptan-3-one | 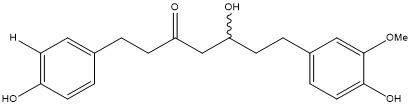 | -7.65 | [25] |  |  |
|  | 2 / mono-ketone | 5-hydroxy-7-(4-hydroxy-3-methoxyphenyl)-1-(4-hydroxyphenyl)heptan-3-one | 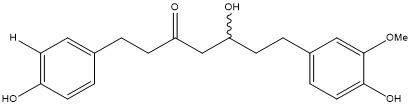 | -7.01 | [25] |  |  |
|  | 2 / mono-ketone | 5-hydroxy-1-(4-hydroxy-3-methoxyphenyl)-7-(4-hydroxyphenyl)heptan-3-one | 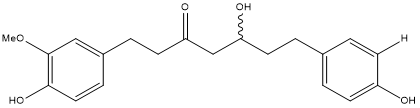 | -8.24 | [25] |  |  |
|  | 2 / mono-ketone | 5-hydroxy-1-(4-hydroxy-3-methoxyphenyl)-7-(4-hydroxyphenyl)heptan-3-one | 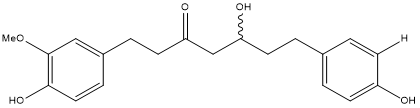 | -7.94 | [25] |  |  |
|  | 2 / mono-ketone | 5-hydroxy-1,7-bis(4-hydroxyphenyl)heptan-3-one | 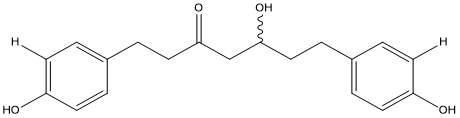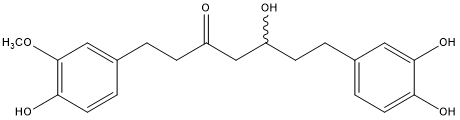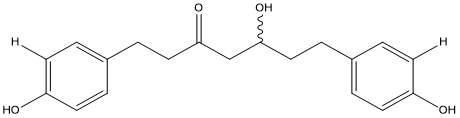 | -8.19 | [25] |  |  |
|  | 2 / mono-ketone | 5-hydroxy-1,7-bis(4-hydroxyphenyl)heptan-3-one |  | -7.12 | [25] |  |  |
|  | 2 / mono-ketone | 7-(3,4-dihydroxyphenyl)-5-hydroxy-1-(4-hydroxy-3-methoxyphenyl)heptan-3-one |  | -8.48 | [21] |  |  |
|  | 2 / mono-ketone | 7-(3,4-dihydroxyphenyl)-5-hydroxy-1-(4-hydroxy-3-methoxyphenyl)heptan-3-one | 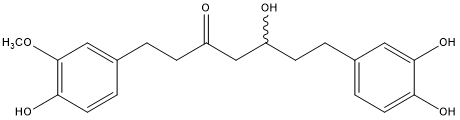 | -7.57 | [21] |  |  |
|  | 2 / mono-ketone | 1-(3,4-dihydroxyphenyl)-5-hydroxy-7-(4-hydroxy-3-methoxyphenyl)heptan-3-one | 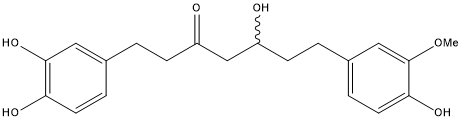 | -9.07 | [21] |  |  |
|  | 2 / mono-ketone | 1-(3,4-dihydroxyphenyl)-5-hydroxy-7-(4-hydroxy-3-methoxyphenyl)heptan-3-one | 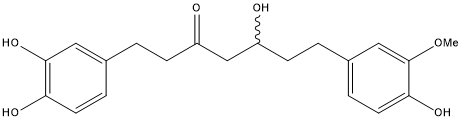 | -7.98 | [21] |  |  |
|  | 2 / mono-ketone | 1,7-bis(3,4-dimethoxyphenyl)-5-hydroxyheptan-3-one | 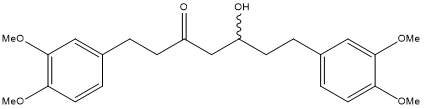 | -7.89 | [21] |  |  |
|  | 2 / mono-ketone | 1,7-bis(3,4-dimethoxyphenyl)-5-hydroxyheptan-3-one | 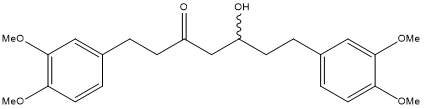 | -4.86 | [21] |  |  |
|  | 2 / mono-ketone | (E)-7-(3,4-dihydroxyphenyl)-1-(4-hydroxy-3-methoxyphenyl)hept-4-en-3-one | 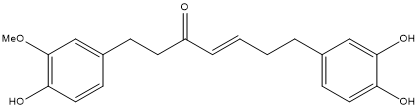 | -8.62 | [21] |  |  |
|  | 2 / mono-ketone | (E)-1-(3,4-dihydroxyphenyl)-7-(4-hydroxy-3-methoxyphenyl)hept-4-en-3-one | 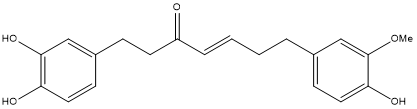 | -7.76 | [21] |  |  |
|  | 2 / mono-ketone | (E)-1,7-bis(3,4-dihydroxyphenyl)hept-4-en-3-one | 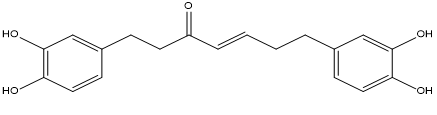 | -8.67 | [21] |  |  |
|  | 2 / mono-ketone | (E)-7-(3,4-dimethoxyphenyl)-1-(4-hydroxy-3-methoxyphenyl)hept-4-en-3-one | 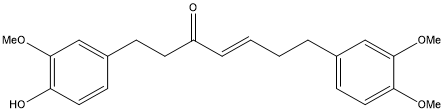 | -7.89 | [21] |  |  |
|  | 2 / mono-ketone | (E)-1-(3,4-dimethoxyphenyl)-7-(4-hydroxy-3-methoxyphenyl)hept-4-en-3-one | 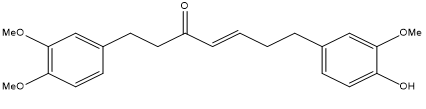 | -7.15 | [21] |  |  |
|  | 2 / mono-ketone | (E)-1,7-bis(3,4-dimethoxyphenyl)hept-4-en-3-one | 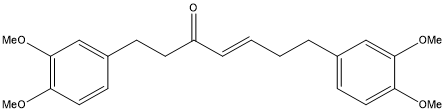 | -5.46 | [21] |  |  |
|  | 2 / mono-ketone | (E)-7-(4-hydroxy-3-methoxyphenyl)-1-(4-hydroxyphenyl)hept-4-en-3-one | 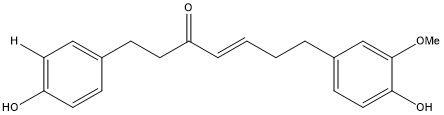 | -7.75 | [21] |  |  |
|  | 2 / mono-ketone | (E)-1-(4-hydroxy-3-methoxyphenyl)-7-(4-hydroxyphenyl)hept-4-en-3-one | 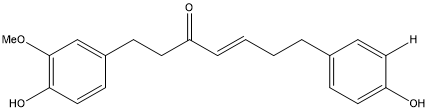 | -7.97 | [21] |  |  |
|  | 2 / mono-ketone | (E)-1,7-bis(4-hydroxyphenyl)hept-4-en-3-one | 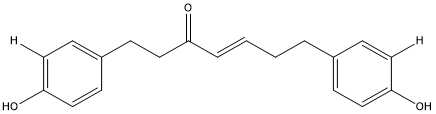 | -6.38 | [21] |  |  |
|  | 2 / mono-ketone | (E)-7-(4-hydroxy-3-methoxyphenyl)-1-(3-methoxy-4-(pentyloxy)phenyl)hept-4-en-3-one | 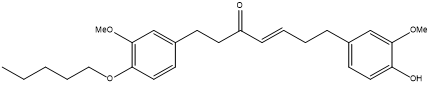 | -5.95 | [21] |  |  |
|  | 2 / mono-ketone | (E)-1-(4-hydroxy-3-methoxyphenyl)-7-(3-methoxy-4-(pentyloxy)phenyl)hept-4-en-3-one | 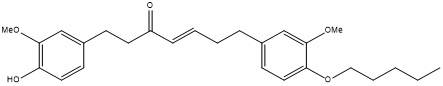 | -5.88 | [21] |  |  |
|  | 2 / mono-ketone | (E)-1,7-bis(3-methoxy-4-(pentyloxy)phenyl)hept-4-en-3-one | 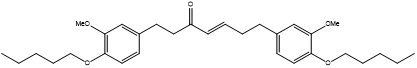 | -6.01 | [21] |  |  |
|  | 2 / mono-ketone | (E)-7-(4-hydroxy-3-methoxyphenyl)-1-(4-(2-hydroxyethoxy)-3-methoxyphenyl)hept-4-en-3-one | 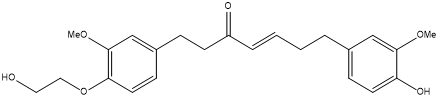 | -8.20 | [21] |  |  |
|  | 2 / mono-ketone | (E)-1-(4-hydroxy-3-methoxyphenyl)-7-(4-(2-hydroxyethoxy)-3-methoxyphenyl)hept-4-en-3-one | 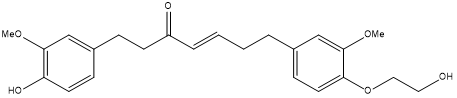 | -7.64 | [21] |  |  |
|  | 2 / mono-ketone | (E)-4-(7-(4-hydroxy-3-methoxyphenyl)-3-oxohept-4-en-1-yl)-2-methoxyphenyl acetate | 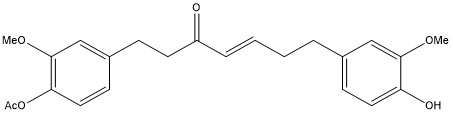 | -6.55 | [21] |  |  |
|  | 2 / mono-ketone | (E)-4-(7-(4-hydroxy-3-methoxyphenyl)-5-oxohept-3-en-1-yl)-2-methoxyphenyl acetate | 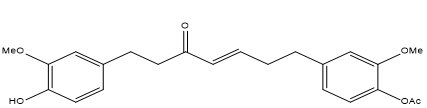 | -6.56 | [21] |  |  |
|  | 2 / mono-ketone | (E)-(5-oxohept-3-ene-1,7-diyl)bis(2-methoxy-4,1-phenylene) diacetate | 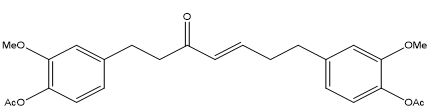 | -4.83 | [21] |  |  |
|  | 2 / mono-ketone | 7-(4-hydroxy-3-methoxyphenyl)-1-(4-hydroxyphenyl)heptan-3-one | 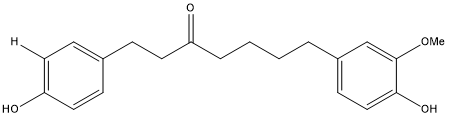 | -7.68 | [21] |  |  |
|  | 2 / mono-ketone | 1-(4-hydroxy-3-methoxyphenyl)-7-(4-hydroxyphenyl)heptan-3-one | 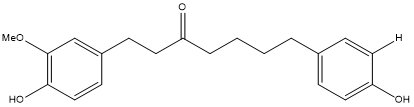 | -7.65 | [21] |  |  |
|  | 2 / mono-ketone | (E)-1,7-diphenylhept-6-en-3-one | 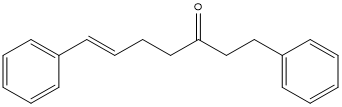 | -7.58 | [26] |  |  |
|  | 2 / mono-ketone | (E)-1-(4-hydroxyphenyl)-7-phenylhept-6-en-3-one | 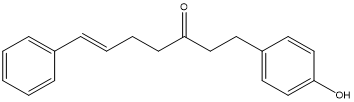 | -5.46 | [26] |  |  |
|  | 2 / mono-ketone | (4E,6E)-1-(4-hydroxyphenyl)-7-phenylhepta-4,6-dien-3-one | 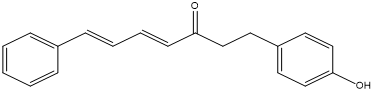 | -8.22 | [26] |  |  |
|  | 2 / mono-ketone | (E)-5-hydroxy-1,7-diphenylhept-6-en-3-one | 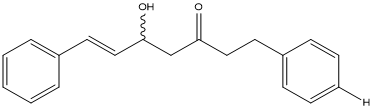 | -8.60 | [24] |  |  |
|  | 2 / mono-ketone | (E)-5-hydroxy-1,7-diphenylhept-6-en-3-one | 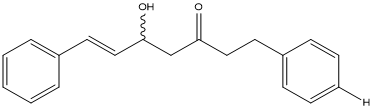 | -7.63 | [24] |  |  |
|  | 2 / mono-ketone | (4E,6E)-1,7-diphenylhepta-4,6-dien-3-one | 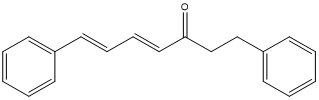 | -8.50 | [24] |  |  |
|  | 2 / mono-ketone | (E)-4-(3-oxo-7-phenylhept-6-en-1-yl)phenyl acetate | 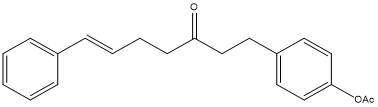 | -7.50 | [24] |  |  |
|  | 2 / mono-ketone | 4-((4E,6E)-3-oxo-7-phenylhepta-4,6-dien-1-yl)phenyl acetate | 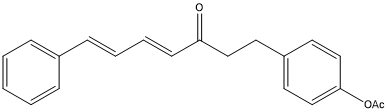 | -7.21 | [24] |  |  |
|  | 2 / mono-ketone | 1,7-diphenylheptan-3-one | 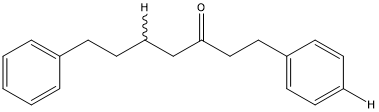 | -5.93 | [24] |  |  |
|  | 2 / mono-ketone | 1-(4-hydroxyphenyl)-7-phenylheptan-3-one | 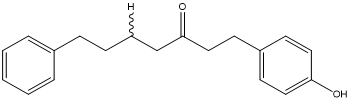 | -6.26 | [24] |  |  |
|  | 2 / mono-ketone | (E)-5-hydroxy-1-(4-hydroxyphenyl)-7-phenylhept-6-en-3-one | 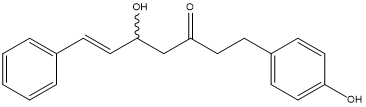 | -6.77 | [24] |  |  |
|  | 2 / mono-ketone | (E)-1-(4-hydroxyphenyl)-7-phenylhept-1-en-3-one | 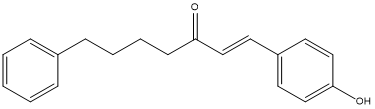 | -6.04 | [24] |  |  |
|  | 2 / mono-ketone | 5-hydroxy-1-(4-hydroxyphenyl)-7-phenylheptan-3-one | 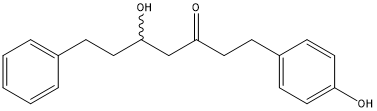 | -7.06 | [24] |  |  |
|  | 2 / mono-ketone | (E)-1-(4-hydroxyphenyl)-7-phenylhept-4-en-3-one | 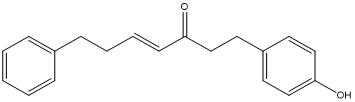 | -5.89 | [24] |  |  |
|  | 3 / pyrazole | 4,4'-((1E,1'E)-(1H-pyrazole-3,5-diyl)bis(ethene-2,1-diyl))bis(2-methoxyphenol) | 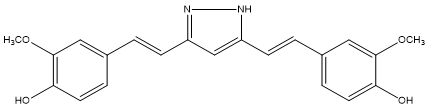 | -8.10 | [22] | AS-KTC014 |  |
|  | 3 / pyrazole | 4-((E)-2-(3-((E)-4-hydroxystyryl)-1H-pyrazol-5-yl)vinyl)-2-methoxyphenol | 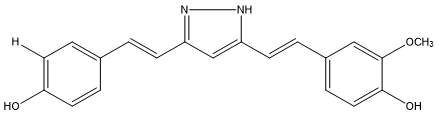 | -7.71 | [22] | AS-KTC015 |  |
|  | 3 / pyrazole | 4,4'-((1E,1'E)-(1H-pyrazole-3,5-diyl)bis(ethene-2,1-diyl))diphenol | 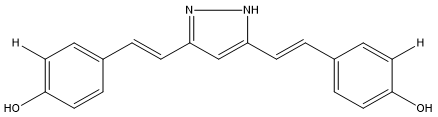 | -7.82 | [22] | AS-KTC016 |  |
|  | 3 / pyrazole | 4,4'-((1E,1'E)-(1-phenyl-1H-pyrazole-3,5-diyl)bis(ethene-2,1-diyl))bis(2-methoxyphenol) | 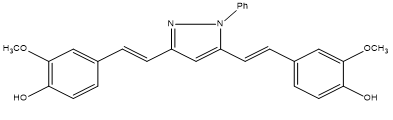 | -8.61 | [22] | AS-KTC017 |  |
|  | 4 / isoxazole | 4,4'-((1E,1'E)-isoxazole-3,5-diylbis(ethene-2,1-diyl))bis(2-methoxyphenol) | 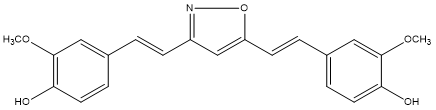 | -8.04 | [22] | AS-KTC018 |  |
|  | 4 / isoxazole | 4-((E)-2-(3-((E)-4-hydroxystyryl)isoxazol-5-yl)vinyl)-2-methoxyphenol | 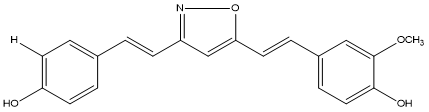 | -7.72 | [22] |  |  |
|  | 4 / isoxazole | 4-((E)-2-(5-((E)-4-hydroxystyryl)isoxazol-3-yl)vinyl)-2-methoxyphenol | 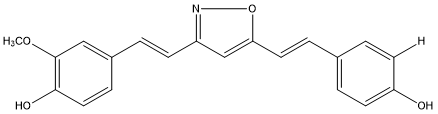 | -8.17 | [22] |  |  |
|  | 4 / isoxazole | 4,4'-((1E,1'E)-isoxazole-3,5-diylbis(ethene-2,1-diyl))diphenol | 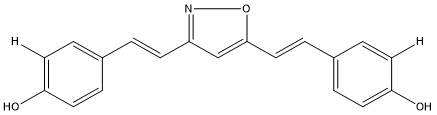 | -7.76 | [22] | AS-KTC020 |  |
|  | 4 / isoxazole | (E)-4-(2-(5-(4-hydroxy-3-methoxyphenethyl)isoxazol-3-yl)vinyl)-2-methoxyphenol | 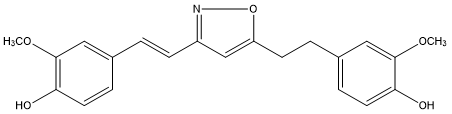 | -7.87 | [22] |  |  |
|  | 4 / isoxazole | (E)-4-(2-(3-(4-hydroxy-3-methoxyphenethyl)isoxazol-5-yl)vinyl)-2-methoxyphenol | 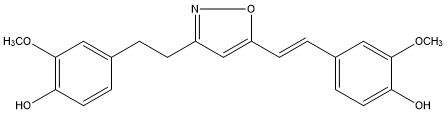 | -7.36 | [22] |  |  |
|  | 4 / isoxazole | 4,4'-(isoxazole-3,5-diylbis(ethane-2,1-diyl))bis(2-methoxyphenol) | 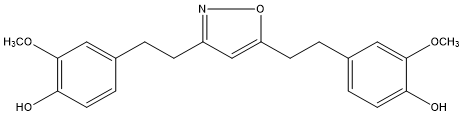 | -7.36 | [22] |  |  |
|  | 4 / isoxazole | 2-methoxy-4-((E)-2-(3-((E)-3-methoxy-4-(pentyloxy)styryl)isoxazol-5-yl)vinyl)phenol | 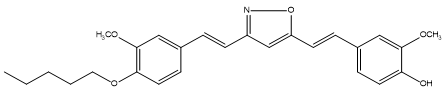 | -7.00 | [22] |  |  |
|  | 4 / isoxazole | 2-methoxy-4-((E)-2-(5-((E)-3-methoxy-4-(pentyloxy)styryl)isoxazol-3-yl)vinyl)phenol | 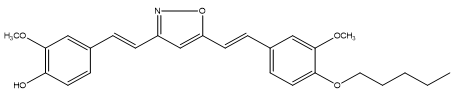 | -6.13 | [22] |  |  |
|  | 4 / isoxazole | 2-methoxy-4-((E)-2-(3-((E)-3-methoxy-4-((3-methylbut-2-en-1-yl)oxy)styryl)isoxazol-5-yl)vinyl)phenol | 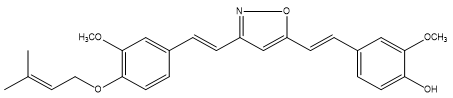 | -7.56 | [22] |  |  |
|  | 4 / isoxazole | 2-methoxy-4-((E)-2-(5-((E)-3-methoxy-4-((3-methylbut-2-en-1-yl)oxy)styryl)isoxazol-3-yl)vinyl)phenol | 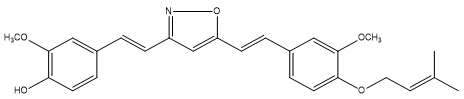 | -7.46 | [22] |  |  |
|  | 4 / isoxazole | 4-((E)-2-(3-((E)-4-ethoxy-3-methoxystyryl)isoxazol-5-yl)vinyl)-2-methoxyphenol | 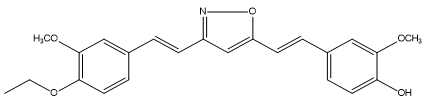 | -7.99 | [22] |  |  |
|  | 4 / isoxazole | 4-((E)-2-(5-((E)-4-ethoxy-3-methoxystyryl)isoxazol-3-yl)vinyl)-2-methoxyphenol | 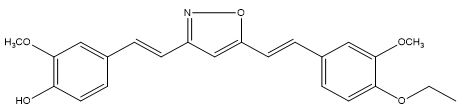 | -7.94 | [22] |  |  |
|  | 4 / isoxazole | 3,5-bis((E)-4-ethoxy-3-methoxystyryl)isoxazole | 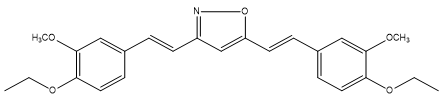 | -7.14 | [22] | AS-KTC022 |  |
|  | 4 / isoxazole | 4-((E)-2-(5-((E)-4-hydroxy-3-methoxystyryl)isoxazol-3-yl)vinyl)-2-methoxyphenyl acetate | 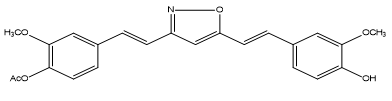 | -7.66 | [22] |  |  |
|  | 4 / isoxazole | 4-((E)-2-(3-((E)-4-hydroxy-3-methoxystyryl)isoxazol-5-yl)vinyl)-2-methoxyphenyl acetate |  | -7.93 | [22] |  |  |
|  | 4 / isoxazole | ((1E,1'E)-isoxazole-3,5-diylbis(ethene-2,1-diyl))bis(2-methoxy-4,1-phenylene) diacetate |  | -7.35 | [22] | AS-KTC023 |  |
|  | 4 / isoxazole | 4-((E)-2-(3-((E)-3,4-dimethoxystyryl)isoxazol-5-yl)vinyl)-2-methoxyphenol |  | -7.76 | [22] |  |  |
|  | 4 / isoxazole | 4-((E)-2-(5-((E)-3,4-dimethoxystyryl)isoxazol-3-yl)vinyl)-2-methoxyphenol |  | -8.27 | [22] |  |  |
|  | 4 / isoxazole | 3,5-bis((E)-3,4-dimethoxystyryl)isoxazole |  | -7.12 | [22] | AS-KTC021 |  |
|  | 4 / isoxazole | 5-((E)-3,4-dimethoxystyryl)-3-((E)-4-methoxystyryl)isoxazole |  | -7.14 | [22] |  |  |
|  | 4 / isoxazole | 3-((E)-3,4-dimethoxystyryl)-5-((E)-4-methoxystyryl)isoxazole |  | -7.45 | [22] |  |  |
|  | 4 / isoxazole | 3,5-bis((E)-4-methoxystyryl)isoxazole |  | -7.33 | [22] | AS-KTC024 |  |
|  | 4 / isoxazole | 4-((E)-2-(3-((E)-4-hydroxy-3-methoxystyryl)isoxazol-5-yl)vinyl)benzene-1,2-diol |  | -8.00 | [22] |  |  |
|  | 4 / isoxazole | 4-((E)-2-(5-((E)-4-hydroxy-3-methoxystyryl)isoxazol-3-yl)vinyl)benzene-1,2-diol |  | -8.32 | [22] |  |  |
|  | 4 / isoxazole | 4,4'-((1E,1'E)-isoxazole-3,5-diylbis(ethene-2,1-diyl))bis(benzene-1,2-diol) |  | -8.67 | [22] | AS-KTC019 |  |
|  | 5 / others | 1,7-bis(4-hydroxy-3-methoxyphenyl)heptane-3,5-diol |  | -8.20 | [22] |  |  |
|  | 5 / others | 1,7-bis(4-hydroxy-3-methoxyphenyl)heptane-3,5-diol |  | -7.76 | [22] |  |  |
|  | 5 / others | (4E,6E)-1,7-diphenylhepta-4,6-dien-3-ol |  | -8.28 | [23] |  |  |
|  | 5 / others | (4E,6E)-1,7-diphenylhepta-4,6-dien-3-ol |  | -8.23 | [23] |  |  |
|  | 5 / others | (E)-1,7-diphenylhept-6-en-3-ol |  | -7.86 | [23] |  |  |
|  | 5 / others | (E)-4,4'-(5-hydroxyhept-3-ene-1,7-diyl)bis(2-methoxyphenol) |  | -7.53 | [21] |  |  |
|  | 5 / others | (S,E)-1,7-diphenylhept-6-en-3-ol |  | -7.03 | [26] |  |  |
|  | 5 / others | (E)-4-(3-hydroxy-7-phenylhept-6-en-1-yl)phenol |  | -7.07 | [26] |  |  |
|  | 5 / others | (E)-4-(3-hydroxy-7-phenylhept-6-en-1-yl)phenol |  | -6.24 | [26] |  |  |
|  | 5 / others | (S,E)-4-(3-hydroxy-7-phenylhept-6-en-1-yl)benzene-1,2-diol |  | -7.41 | [26] |  |  |
|  | 5 / others | (R,4E,6E)-1,7-diphenylhepta-4,6-dien-3-ol |  | -8.28 | [26] |  |  |
|  | 5 / others | (E)-1,7-diphenylhept-6-en-3-yl acetate |  | -7.88 | [24] |  |  |
|  | 5 / others | (E)-1,7-diphenylhept-6-en-3-yl acetate |  | -7.60 | [24] |  |  |
|  | 5 / others | (E)-1,7-diphenylhept-6-en-3-ol |  | -7.88 | [24] |  |  |
|  | 5 / others | (E)-1,7-diphenylhept-6-en-3-ol |  | -6.90 | [24] |  |  |
|  | 5 / others | (E)-4-(3-hydroxy-7-phenylhept-6-en-1-yl)benzene-1,2-diol |  | -7.65 | [24] |  |  |
|  | 5 / others | (E)-4-(3-hydroxy-7-phenylhept-6-en-1-yl)benzene-1,2-diol |  | -5.61 | [24] |  |  |
|  | 5 / others | (1E,3E,5E)-hepta-1,3,5-triene-1,7-diyldibenzene |  | -8.54 | [24] |  |  |
|  | 5 / others | (S,E)-1-(4-methoxyphenyl)-7-phenylhept-6-en-3-ol |  | -6.91 | [24] |  |  |
|  | 5 / others | (R,E)-1-(4-methoxyphenyl)-7-phenylhept-6-en-3-ol |  | -6.25 | [24] |  |  |
|  | 5 / others | (R,4E,6E)-1,7-diphenylhepta-4,6-dien-3-yl acetate |  | -7.94 | [24] |  |  |
|  | 5 / others | (S,E)-1,7-diphenylhept-6-en-3-yl acetate |  | -7.56 | [24] |  |  |
|  | 5 / others | (S)-1,7-diphenylheptan-3-ol |  | -5.76 | [24] |  |  |
|  | 5 / others | (S)-1,7-diphenylheptan-3-yl acetate |  | -7.36 | [24] |  |  |

|  |  |  |  |  |  |  |  |  |
| --- | --- | --- | --- | --- | --- | --- | --- | --- |
|  | **Supplementary Table 2** Explanation of each simulation steps of minimization and molecular dynamics simulations. | | | | |  |  |  |
|  | Step | Restraint mask | Meaning | Constraint force (kcal/mol) | maxcycle/ncycle | Simulation time (ps) | tempi (K) | temp0 (K) |
|  | min1 | 706-993 except H & water | restraints on the protein structure, heavy atoms | 5 | 10,000/5,000 | - | - | - |
|  | min2 | 706-993 backbone | restraints on the protein structure, backbone atoms: C, CA, O, N | 5 | 10,000/5,000 | - | - | - |
|  | min3 | 706-993 backbone |  | 1 | 10,000/5,000 | - | - | - |
|  | min4 | 706-993 backbone |  | 0.5 | 10,000/5,000 | - | - | - |
|  | min5 | - | the entire system was minimized with no positional restraints | - | 10,000/5,000 | - | - | - |
|  | md1 | 706-993 allatom | heat the systems up with NVT ensemble | 5 | - | 200 | 0 | 300 |
|  | md2 | - | NVT ensemble equilibration | - | - | 500 | 300 | 300 |
|  | md3 | - | NPT ensemble, the production runs | - | - | 30000 | 300 | 300 |
|  |  |  |  |  |  |  |  |  |

|  | **Supplementary Table 3** List of binding residues of each system, which mentioned in Figure 3 | | | |
| --- | --- | --- | --- | --- |
|  | SYR-HER2TK | AS-KTC006-HER2TK | AS-KTC021-HER2TK |  |
|  |  |  |  |  |
|  | Leu726 | Leu726 | Leu726 |  |
|  | Gly727 | Gly727 | Gly727 |  |
|  | Ser728 | Ser728 | Ser728 |  |
|  | Gly729 | Gly729 | Gly729 |  |
|  | Ala730 | Val734 | Val734 |  |
|  | Val734 | Ala751 | Ala751 |  |
|  | Ala751 | **Lys753** | **Lys753** |  |
|  | Ile752 | **Glu770** | **Glu770** |  |
|  | **Lys753** | Ala771 | Ala771 |  |
|  | Leu755 | Met774 | Met774 |  |
|  | **Glu770** | Ser783 | Ser783 |  |
|  | Ala771 | Arg784 | Arg784 |  |
|  | Met774 | Leu785 | Leu785 |  |
|  | Ser783 | Leu796 | Leu786 |  |
|  | Arg784 | Thr798 | Leu796 |  |
|  | Leu785 | Gly804 | Val797 |  |
|  | Leu796 | **Cys805** | Thr798 |  |
|  | Val797 | Asp808 | Gly804 |  |
|  | Thr798 | Arg849 | **Cys805** |  |
|  | Gln799 | Asn850 | Leu807 |  |
|  | Leu800 | Leu852 | Asp808 |  |
|  | **Met801** | *Thr862* | Arg849 |  |
|  | Gly804 | *Asp863* | Asn850 |  |
|  | **Cys805** | *Phe864* | Leu852 |  |
|  | Arg849 | Gly865 | *Thr862* |  |
|  | Asn850 |  | *Asp863* |  |
|  | Leu852 |  | Ala867 |  |
|  | *Thr862* |  |  |  |
|  | *Asp863* |  |  |  |
|  | *Phe864* |  |  |  |
|  |  |  |  |  |
|  |  |  |  |  |
|  | bold&underline = salt bridge residues | |  |  |
|  | bold = H-bonding residue(s) | |  |  |
|  | italic&underline = DFG motif residues | |  |  |
